# Editorial: New insights into neurodevelopmental biology and autistic spectrum disorders

**DOI:** 10.3389/fnins.2024.1514075

**Published:** 2025-01-07

**Authors:** Michela Candini

**Affiliations:** Department of Psychology “Renzo Canestrari” University of Bologna, Bologna, Italy

**Keywords:** autism spectrum disorder, heterogeneity, genetic, phenotypes, neurobiological mechanism

Autism spectrum disorder (ASD) refers to a broad range of neurodevelopmental conditions characterized by challenges with social skills, repetitive behaviors, speech and nonverbal communication (American Psychiatric Association, 2013). Autistic symptoms emerge in early childhood and persist throughout lifetime (Christensen et al., 2016). A core feature of ASD manifestations concerns its heterogeneity in terms of onset, comorbidities, behavioral expression, and treatment-response, along with heterogeneous genetic and neurobiological underpinnings (Lombardo et al., 2019). Pioneering research focused on the genetic, environmental, and neurodevelopmental biological factors may help understand the broader range of phenotype manifestations observed in ASD population. In this frame, clarifying how the brain phenotypes determine specific social and cognitive profiles could provide valuable insights for clinicians, aiding in the translation of research findings into clinical practice, and supporting the implementation of tailored interventions.

From these statements emerges the inspiring idea to create the present Research Topic, a collection of the more recent cutting-edge contributions unraveling important insights on the neurobiological and genetic features of ASD. Finally, the current Research Topic consists of 11 papers (one Review paper, two Hypothesis and Theory papers, and eight Original Research). In this editorial, I will discuss different themes: (i) brain anatomical differences reported in ASD and their association with clinical phenotype; (ii) genetic variations and biological implications to pathogenesis of ASD from both animal and human studies. A final discussion on future directions is presented.

In the last decades, multiple and subtle brain structural alterations appear to be involved in ASD symptomatology. These anatomical changes include atypical cortical thickness (Hardan et al., 2006; Hyde et al., 2010), increased gray matter volume (Retico et al., 2016; Lucibello et al., 2019), altered brain structure asymmetry (Gage et al., 2009; Floris et al., 2016; Postema et al., 2019) as well as microstructural connectivity disruptions (Cheon et al., 2011; Ameis and Catani, 2015). Weber et al. evaluated the effects of age on white matter microstructural disintegrity by examining Diffusion Tensor Imaging (DTI) metrics and connectome Edge Density in a large dataset of ASD and control patients, representing different age cohorts. The authors showed age-dependent ASD-related changes which become evident in adolescents and young adults, but not in infants and toddlers. Parekh et al. focused on hemispheric asymmetry associated with sensory processing dysfunction, which is now considered a diagnostic hallmark of ASD. The authors compared white matter tract asymmetry for those children with vs. without sensory over-responsivity (SOR) using the more advanced NODDI technique. Differences in the degree of lateralization of specific tracts for children with SOR compared to those without SOR were found, suggesting that this neurodevelopmental phenotype may have a laterality signature. Furthermore, Li et al. showed that the gray matter asymmetries observed in specific brain regions are selectively associated with the three core symptoms of ASD: social interaction, verbal communication and restricted repetitive behaviors.

To explore the relationship between the level of CSF and the development of ASD, Sotgiu et al. adopted an innovative neuroimaging tool that allows to segment and quantify perivascular spaces in the white matter (WM-PVS). The authors found that WM-PVS dilation can be considered a neuroimaging marker of male ASD individuals, especially the youngest and the more severe ones.

Adopting a neurodevelopmental perspective, Xiong et al. reviewed the main functions of microglia and astrocyte cells in the developing brain and their contributions to ASD. Glial cells are indispensable regulators of inflammatory responses, synaptic function, and plasticity, and seem to be involved in the pathogenesis of ASD. To go further in understanding this Research Topic, Carroll et al. described the role of microglia heterogeneity in the development of multisensory midbrain in neonatal mice. Animal models are instrumental in enhancing our understanding of the neurocognitive processes affected in neurodevelopmental disorders, such as ASD, by mimicking certain genetic and neurobiological features.

As far as genetic variations and neurobiological implications to neurodevelopment, recent evidence demonstrated that CAMK2G is one of the main CAMK2 isozymes expressed during early neurodevelopment, both in humans and animals. A mutation in this gene causes a neurodevelopmental disorder (Proietti Onori et al., 2018). Here, Rigter et al. obtained global Camk2g knockout mice and performed an extensive phenotypic characterization revealing that this protein plays a key role in motor and innate behavior, and further supporting its role in the developing brain.

Deletion and duplication of the human 16p11.2 locus are found in patients with autism spectrum disorders, intellectual disability and other psychiatric traits. However, the high gene density associated with the region and the strong phenotypic variability makes the study of the 16p11.2 syndromes extremely complex. Martin Lorenzo et al. studied the social deficits caused by 16p11.2 alterations in two rat outbred strains. The rat models displayed defects in social behavior coherent with evidence coming from mice and human studies, suggesting that these rat models may help in understanding the brain mechanisms devoted to social behavior in humans.

Recent evidence reveals that maternal inflammation or immune activation influences RNA epitranscriptomic mechanisms, resulting in an altered fetal brain development. Beopoulos et al.(a) described the mRNA epitranscriptomic mechanisms, which function cooperatively and could, in association with both genotypes and environmental conditions, alter spatiotemporal proteins expression patterns during brain development. RNA epitranscriptomics might probably take precedence over differential epigenetic (methylation/acetylation) modifications which require environmental conditions to stay stable long enough to have real physiological effects. Thus, epitranscriptomics dysregulation can be considered a major determinant for the significantly increased risk of ASD pathogenesis.

Nowadays, the precise role of environmental mutagenesis in neurodevelopmental disorders etiology is under debate. Here, Baker et al. explored environmental mutation vulnerability of disease-associated gene sets revealing that environmental mutagenesis, including radiation and polycyclic aromatic hydrocarbons, disproportionately mutate genes related to neurodevelopmental disorders including ASD, schizophrenia and ADHD.

To conclude, I would like to mention the paper by Beopoulos et al.(b) which provides a comprehensive model of the ASD pathogenesis. The model here proposed is rooted in the very early neurogenesis stage and neuronal migration and highlights the conditions and developmental window where alterations in neural development can have major effects on a wide range of core symptoms.

The present Research Topic offers an extensive overview of the rich landscape of research on the neurobiology of ASD, focusing on brain structural and genetic alterations associated with ASD. Moreover, this Research Topic underscores the complexity of conducting research in the field of ASD due to the neurobiological heterogeneity and complex phenotypic expression associated with this condition. Thus, I hope that the present studies contribute to stimulate further research toward still open questions and novel challenges that can potentially increase the autistic community wellbeing. I wish to warmly thank the authors and the reviewers that contributed to this Research Topic.
